# Tannin-Mordant Coloration with Matcha *(camelia sinensis)* and Iron(II)-Lactate on Human Hair Tresses

**DOI:** 10.3390/molecules26040829

**Published:** 2021-02-05

**Authors:** Lusine Sargsyan, Thomas Hippe, Hartmut Manneck, Volkmar Vill

**Affiliations:** 1 R&D Hair Color Level 3, Henkel AG & Co KGaA, Hohenzollernring 125-127, 22763 Hamburg, Germany; Thomas.Hippe@henkel.com (T.H.); Hartmut.Manneck@henkel.com (H.M.); 2 Department of Chemistry, University of Hamburg, Martin-Luther-Kingplatz 6, 20146 Hamburg, Germany; vill@chemie.uni-hamburg.de

**Keywords:** tannin-mordant-complex, natural hair coloration, matcha, ΔE-values, wash fastness

## Abstract

The aim of this work was to optimize our natural hair dyeing system which we described in our previous work and to compare with other dyeing systems. Therefore, we investigated concentration limits of matcha and mordant and compared this new dyeing method with commercial permanent systems on the market. Completely unpigmented hair tresses were dyed with matcha powder *(camelia sinensis)* and iron(II)-lactate. To investigate the wash fastness and concentration limits, the differently dyed hair tresses were spectrophotometrically measured. The comparison of the damage potential for which cysteic acid is an indicator was measured by NIR. The concentration of matcha and mordant are responsible for the intensity of the color results. The higher the matcha or the mordant concentration, the darker the color results of the dyed hair tresses. Hair damage of matcha mordant dyeing is comparable with results of commercial permanent hair coloration systems. Moreover, the results of wash fastness of matcha mordant dyed hair tresses is comparable and even better by tendency to permanent colored hair tresses.

## 1. Introduction

One of the most desirable goals in the hair dye industry is to develop a color that will permanently dye the hair without damaging, while still providing long-lasting color intensity. The interest in natural and renewable raw materials as an alternative source has increased in many areas. [1] Natural hair dyeing colorants such as henna are widely used in natural cosmetics. The main coloring agent in henna leaves is lawsone (2-hydroxy-1,4-naphthoquinone), also known as CI Natural Orange 6, which is responsible for the coloration of the hair fibers and leads to red-orange color results. [2,3].

Natural colorants such as iron-gall inks have been used for thousands of years. They represent a colored tannin-iron complex. They have been used to dye leather, fabrics, and other materials, whereby different plants provide different tanning agents, thus creating a wide variety of colors [4].

There are many categories of synthetic hair dyes, which are differentiated by their ability to penetrate the surface or deeper parts of the hair shaft and the longevity of the color after washing. The two most used types of hair dyes are temporary and permanent colorations. Temporary dyes damage the hair less than permanent dyes and are not capable to reach the cortex. [3] The lastingness of this intensive colors is limited to 5–8 hair washes.

An alternative method instead of henna and synthetic coloration could be a dyeing system with polyphenolic compounds of natural origin and mordants with other color results than reddish ones.

While the polyphenolic compound itself has only a low substantivity to the hair fiber, it is necessary to use metal salts as mordants to increase the affinity by formation of stable colored complexes. [5] Iron salts, especially iron(II)-lactate, form intensive coloration in combination with polyphenolic compounds in comparison to other metal salts as investigated in our previous work [6].

Tannic substances can be extracted from different parts of plants as barks and leaves and also from tea to use it for hair dyeing methods. [5] All investigations in this work were done with matcha tea powder *(camelia sinensis)* itself because of the tannic and catechine compounds which are present in the powder.

In our previous work, we could achieve intensive coloration on pre-bleached hair tresses. [6] In this current work, the investigations were carried out on unpigmented hair tresses to determine whether intensive color results can also be achieved on untreated hair tresses. In addition, the wash fastness and the damage potential were compared to commercially available permanent dyeing systems. The examination of hair tresses using near-Infrared (NIR)-spectroscopy has become an established method regarding the detection of cysteic acid. For instance, Pande and Yang examined hair tresses damaged by oxidative treatment using NIR spectroscopy. [7]. Miyamae et al. applied partial least squares (PLS) regression to NIR-DR spectra of human hair after mean centering (MC), standard normal variate (SNV), and first derivative (1d) or second derivative (2d) analysis to develop calibration models that predict physical properties of human hair [8]. Thus, the cysteic acid determination in our studies was also carried out by NIR spectroscopy and determined by creating a calibration set and a validation set.

## 2. Results and Discussion

### 2.1. Investigation of the Concentration of Matcha

**Figure 1:**
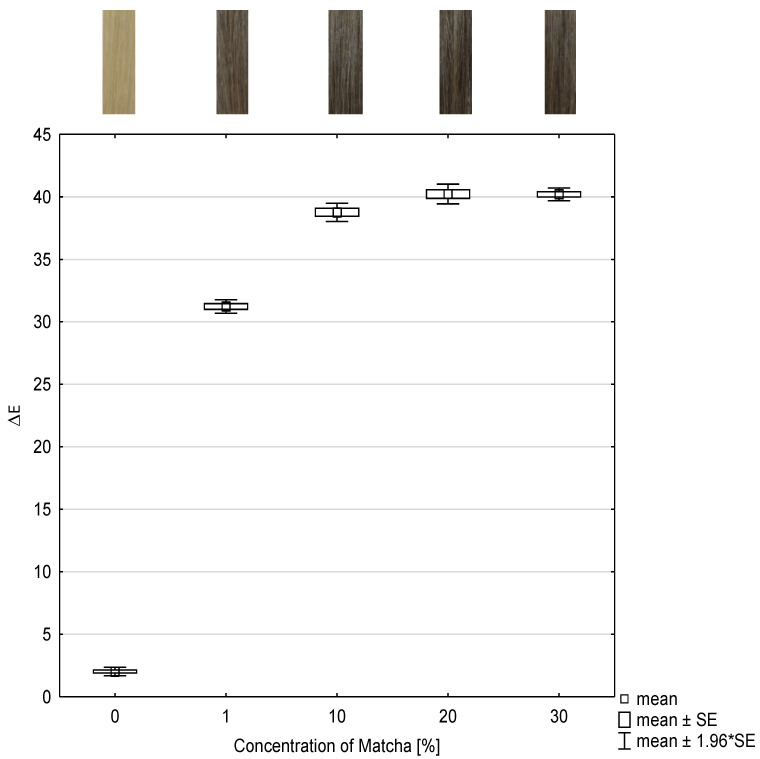
Box and whisker plot of color change ΔE-value of dyed hair tresses with different concentrations of matcha in combination with 1 wt% iron(II)-lactate and the corresponding photographs of the hair tresses (n = 10). SE: standard error.

To investigate the dependence of the concentration of the tanning agent, herein matcha, and the color intensity, the color change values were determined and presented as box plots in Figure 1. The abscissa represents the concentration of matcha and the ordinate represents the color change as ΔE-value after each treatment. The ΔE-value describes absolute color differences calculated by the Euclidean color distance formula as described in Section 4.5 in Formula (1). Moreover, the color coordinates L, a, b, and ΔE are listed in Table 1.

Compared to the reference, which is the untreated hair tress, all ΔE-values of the hair tresses treated with 1–30 wt% matcha and then treated with mordant differ statistically significantly with (*p* ≤ 0.001).

No more significant differences (*p* = 1) are identifiable between the ΔE-values of hair tresses dyed with 20 wt% and 30 wt% matcha and then with 1 wt% iron(II)-lactate.

Despite significant differences with (*p* ≤ 0.001) between hair tresses, which where dyed with 10 wt% and 20 wt% matcha and afterwards with 1 wt% iron(II)-lactate, the ΔE-values only raise low.

Higher concentration of matcha tends to result in more intense colors. From these investigations, it is evident that the color intensity correlates with the concentration of matcha. An optimal color result can be expected at a concentration of 10 wt% matcha or more.

### 2.2. Investigation of the Concentration of Iron(II)-Lactate

**Figure 2:**
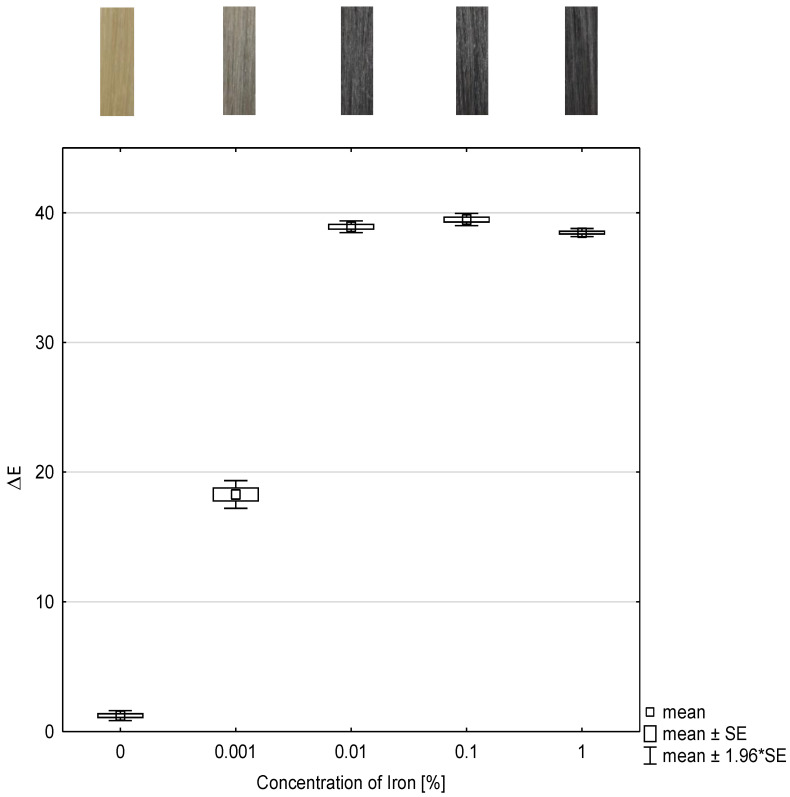
Box and whisker plot of color change ΔE-value of dyed hair tresses with different concentrations of iron(II)-lactate in combination with 10 wt% matcha and the corresponding photographs of the hair tresses (n = 10). SE: standard error.

To investigate the dependence of the concentration of the mordant, herein iron(II)-lactate, and the color intensity, the color change values were determined and presented as box plots in Figure 2. The abscissa represents the concentration of iron(II)-lactate and the ordinate represents the color change as ΔE-value after each treatment. Moreover, the color coordinates L, a, b, and ΔE are listed in Table 2.

As in the study of the concentration of the tanning agent, Figure 2 also shows that all ΔE-values of the treated hair tresses differ statistically significantly with (*p* ≤ 0.001) from the ΔE-value of the untreated hair tress, which is the reference.

The delta ΔE-values of hair tresses treated with 0.01 wt% iron(II)-lactate and matcha do not show statistically significant differences (*p* = 1) compared to the ΔE-values treated with 0.1 wt% or 1 wt% iron(II)-lactate.

Intensive color results can be achieved at concentrations as low as 0.01 wt% iron(II)-lactate. From these investigations, it is evident that the color intensity correlates with the concentration of iron(II)-lactate as mordant. An optimal color result can be expected at a concentration of 0.01 wt% of iron(II)-lactate or more.

### 2.3. NIR-Measurement of Cysteic Acid Amount of Matcha and Mordant and Dove Grey Dyed Hair Tresses

**Figure 3:**
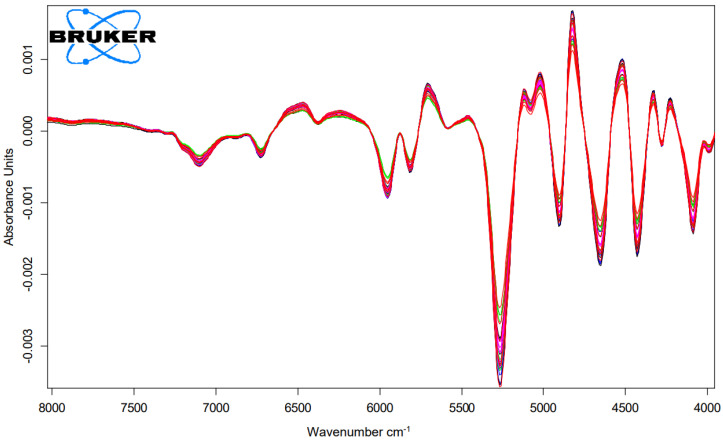
First derivation of FT-NIR-spectra of hair tresses after 3 times bleaching and dyed with matcha and iron-(II)-lactate with n = 5.

**Figure 4:**
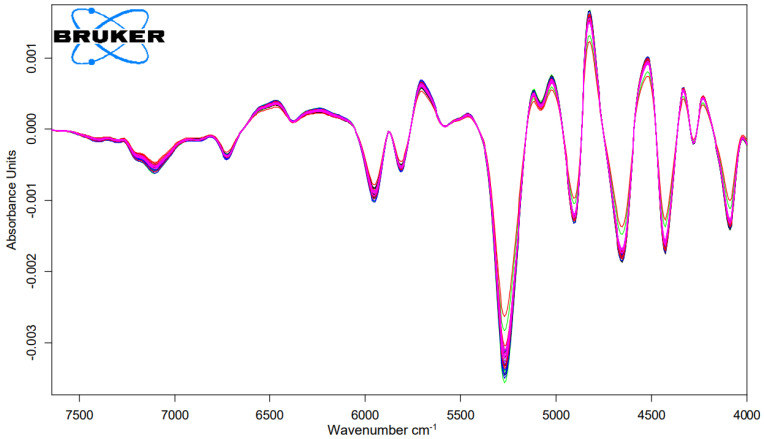
First derivation FT-NIR-spectra of hair tresses after 3 times bleaching and dyed with Dove Grey with n = 5.

**Figure 5:**
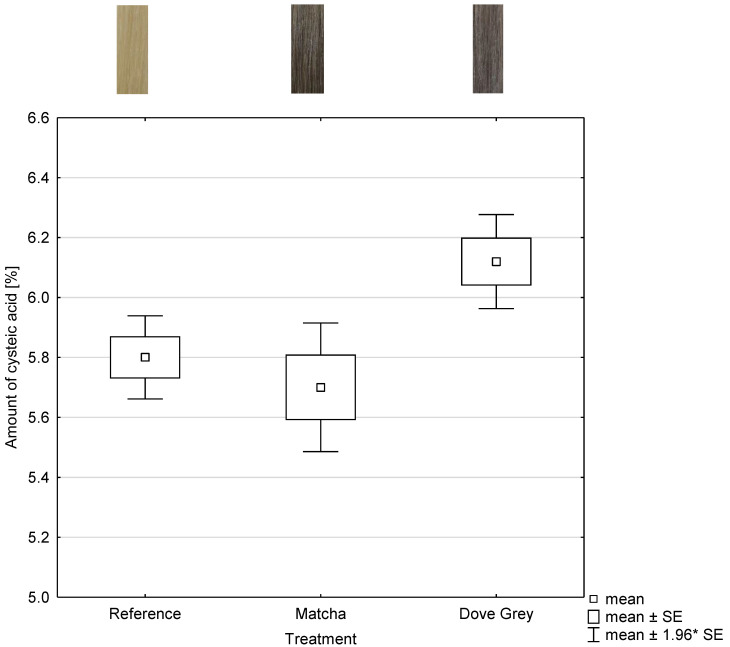
Box and whisker plot of comparison to untreated hair tresses as reference of the amount of cysteic acid amount of hair tresses treated with 10 wt% matcha in combination with 1 wt% iron(II)-lactate and hair tresses dyed with Dove Grey with the corresponding photographs of the hair tresses (n = 5). SE: standard error.

To obtain information on the damage potential of the tanning and mordant dyeing, the cysteic acid amount was determined using NIR measurements. The relevant NIR-range for the analysis were the range of the wavelength 7300 to 4020 cm^−1^ as shown in Figure 3 and Figure 4. The abscissa represents each treatment and the ordinate represents the amount of cysteic acid after each treatment as shown in Figure 5.

The product of oxidative treatments is cysteic acid, which is an indicator of damage to the hair structure. The higher the cysteic acid content, the more serious the damage [9]. No statistical differences (*p* = 1) can be observed between the 3 times bleached hair tress, which is the reference, and both dyed hair tresses. A higher number of samples or a confidence level of 10 wt% would probably lead to significant differences between the cysteic acid amount of the reference and the cysteic acid amount of Dove Grey (Igora Royal Absolutes Silverwhite, Schwarzkopf) treated hair tresses. Statistical differences with (*p* ≤ 0.001) between the cysteic acid amount of matcha and iron(II)-lactate dyed and Dove Grey treated hair tresses are visible. The cysteic acid amount of Dove Grey dyed hair tresses is higher by tendency as shown in Figure 5.

The potential to damage the hair is low or almost non-existent in both treatments. Thus, the hair coloration by using tanning agents and mordants is comparable and even better by tendency to a permanent hair coloration system.

### 2.4. Wash Fastness of Matcha and Mordant and Dove Grey Dyed Hair Tresses

**Figure 6:**
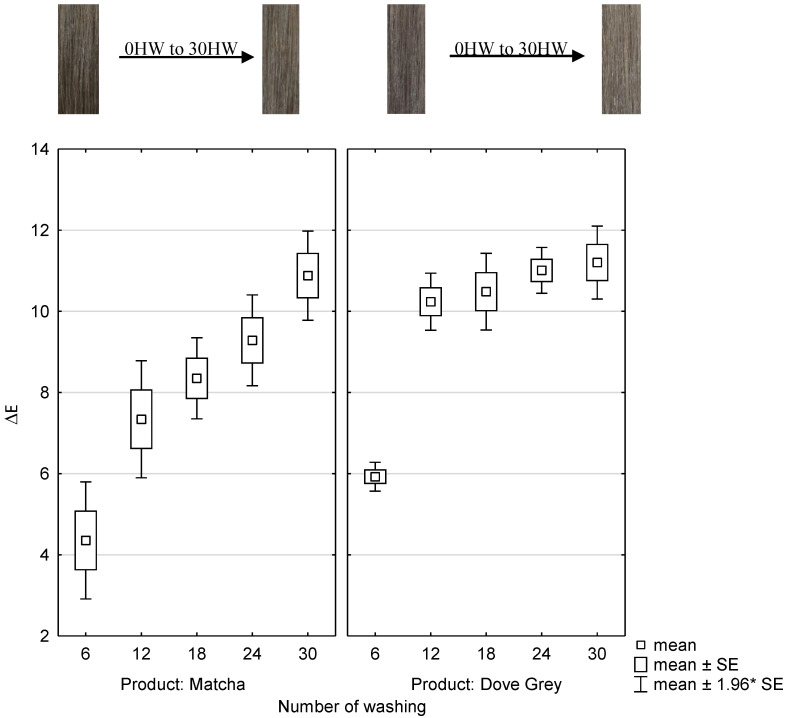
Box and whisker plots of color change ΔE—values after washing numbers of 6 to 30 of dyed hair tresses with 10 wt% matcha in combination with 1 wt% iron(II)-lactate compared to hairs tresses dyed with Dove Grey and the corresponding photographs of the hair tresses from the reference 0 hair washes to 30 hair washes (n = 5). SE: standard error.

To test the wash fastness, matcha and iron(II)-lactate treated hair tresses and Dove Grey dyed hair tresses were hand washed up to 30 times and measured spectrophotometrically. The abscissa represents each number of hair washing (HW) and the product which was used for the treatment. The ordinate represents the ΔE-values after of each treatment after the number of hair washings as shown in Figure 6. Moreover, the color coordinates L, a, b, and ΔE are listed in Table 3 and Table 4.

It can be observed that the removal of hair color is even when the hair tresses are dyed with matcha and iron(II)-lactate. When coloring with matcha and iron(II)-lactate, the ΔE-values of the hair tresses, which were washed up to 6 times, differ statistically significant with (*p* ≤ 0.001) from all other hair washes up to 30 times.

Significant differences with (*p* ≤ 0.001) can be noticed between the ΔE-values of the hair tresses which were washed up to 12 times and the hair tresses washed up to 30 times after dyed with matcha and iron(II)-lactate. Therefore, the color will not change statistically significant after more than 12 hair washes, when hair tresses are dyed with matcha and iron(II)-lactate.

When coloring with Dove Grey, the ΔE-values of the hair tresses, only which were washed up to 6 times, differ statistically significant with (*p* ≤ 0.001) from all other hair washes up to 30 times. Therefore, the color will not change statistically significant after more than 6 hair washes, when hair tresses are dyed with Dove Grey.

The ΔE-values of matcha and iron(II)-lactate dyed hair tresses and Dove Grey dyed hair tresses are comparable after 30 hair washes.

This means that the new dyeing system with matcha and iron(II)-lactate can also keep up with the tests of wash fastness with a permanent hair dye as Dove Grey.

### 2.5. Possible Chemical Bonds on the Hair Fibre after Tannin and Mordant Dyeing

According to the investigations of Covington A. D., the protein tannin interactions are based on various types of bonds as hydrogen bonds, salt links between amino side chains and carboxylic acid groups, or hydrophobic interactions [10].

**Figure 7:**
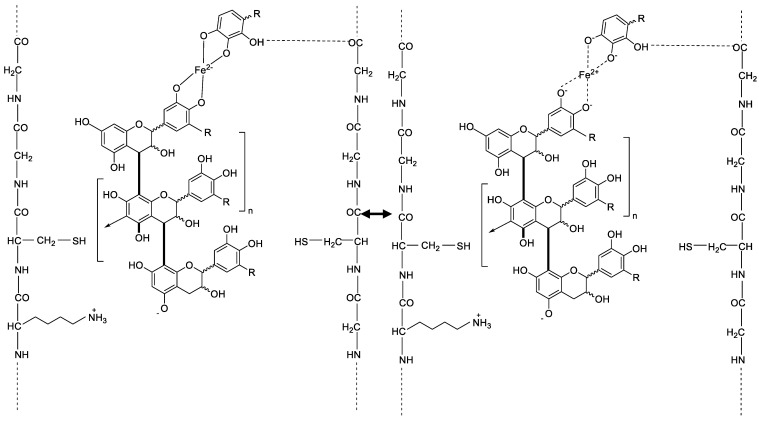
Possible interactions between hair fibers and flavonol compounds of matcha and the mesomeric effect of iron(II)-lactate where R=-H, -CH, or -OH.

The following Figure 7 shows possible bonds and interactions of the hair fiber with ingredients of matcha, here a flavonoid, and iron. The mesomeric effect and the complex bonds that can be formed from the iron ion to the hydroxy groups of the flavonols are shown. It can be seen that the iron can be in a boundary state as an anion covalently bound with the charge −3 or coordinately bound with the charge +3. Furthermore, Figure 7 shows the ionic bond between a negative charged oxygen of the flavonol and a positive charged amino side chain group. Numerous hydrogen bonds can also be formed between the hydroxy groups of flavonol and keratinous hair structure.

## 3. Conclusions

These studies show the potential of the hair dye system developed here to compete in the hair dye industry. The investigations of the damage potential and the wash fastness with a permanent coloring product on the market show that this system provides comparable and better results. To confirm our obtained results, further investigations are necessary. For example, other parameters can also be investigated before in vivo experiments can be performed. In addition, the exposure times can still be optimized, and the sequence of the procedure can be varied. Further investigations with different tanning agents and mordants should be carried out to generate additional color shades.

## 4. Materials and Methods

Hair tresses were obtained from the leading manufacturer Kerling International Haarfabrik GmbH (Backnang, Germany) with a total length of 10 cm of which 8 cm include hair fibers. Completely unpigmented hair tresses from Caucasians with 0.45 ± 0.05 g weight, excluding glue bond, are used for the investigations in this work.

### 4.1. Application of Permanent Coloration

To compare vegetable tannin coloration with commercially available permanent hair color for professional hairdressing needs, Igora Royal Absolutes Silverwhite with the color direction Dove Grey (Schwarzkopf Professional, Düsseldorf, Germany) was used. Ratio of color cream to developer is 1:1 with an application time of 20 min and 3% developer. After mixing of developer and color cream 9 g for one hair tress was used.

### 4.2. Method of Matcha and Mordant Dyeing

**Figure 8:**
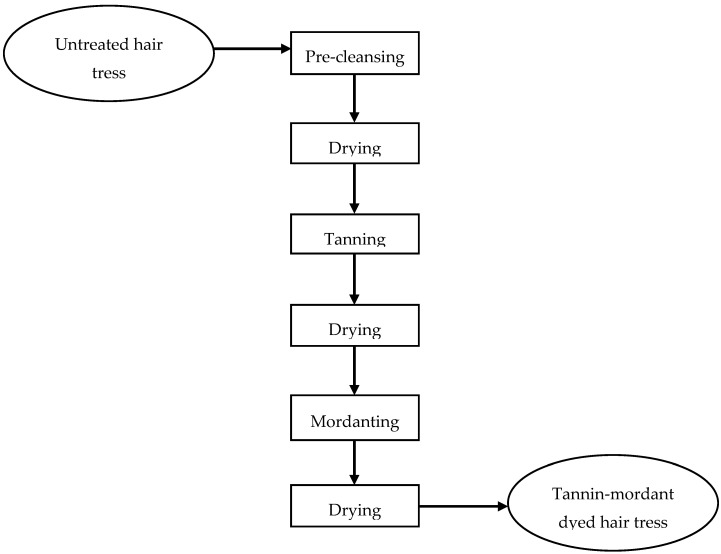
Flowchart of complete dyeing method.

All investigations according to mordant coloration where done with matcha tea powder and iron(II)-lactate. The matcha tea used in this work was obtained from The Tea Company GmbH & Co. KG (Neu Wulmstorf, Germany) with 100% origin in Japan and the Article No. 174077. Tencha (camellia sinensis) is the origin product of which the matcha powder is manufactured by stonegrounds. [11] As shown in Figure 8, the flowchart of the complete tannin-mordant dyeing method is listed and will be described more precise in the following. Matcha solutions of 1 wt%, 10 wt%, 20 wt%, and 30 wt%, whereby 1 wt% matcha solution contains about 0.3 wt% polyphenols, were prepared in a buffer system of pH = 5. (Gentle to the hair, as the ISP of the hair is about pH 4.6). The hair tresses were each treated for 30 min in the respective solution (100 mL/g) and then rinsed under running deionized water for 1 min under 20 times combing. Afterwards, the hair braids were dried with a commercially available hair dryer at a defined distance and temperature (T = 80 ± 5 °C; d = 10 cm) under 20-fold combing.

Subsequently, 0.001–1 wt% metal salt solutions were prepared. The tannin-treated hair tresses were each treated for 30 min in the respective solution (100 mL/g) and then rinsed under running deionized water for 1 min under 20-fold combing. Afterwards, the hair tresses were dried with a commercially available hair dryer at a defined distance and temperature (T = 80 ± 5 °C; d = 10 cm) under 20-fold combing.

### 4.3. Method of Wash Fastness

The hair strands were washed under deionized running water with a volume flow of V = 40 ± 20 mL/s (T = 33 ± 2 °C) for 1 min to remove coarse contaminants first. To review the wash fastness of the color, the wet hair tresses were washed up to 30 times by hand using 0.5 ± 0.02 g/hair commercially available shampoo (Schauma 7 Kräuter, Schwarzkopf). The 7 Herbs Shampoo was massaged in 10 times with a 5-fold circular motion with the thumb and forefinger and constant pressure from the adhesive bond to the tip of the hair. After incorporation of the shampoo, the hair tresses were rinsed out under running deionized water under deionized running water with a volume flow of V = 40 ± 20 mL/s (T = 33 ± 2 °C) for 1 min. After rinsing, the hair tresses were detangled by combing them 20 times, starting from the adhesive bond up to the tip of the hair. After cleaning and combing, the hair tresses were dried with a commercially available hair dryer at a defined distance and temperature (T = 80 ± 5 °C; d = 10 cm). This process was repeated up to 30 hair washes.

### 4.4. Cysteic Acid Measurement with NIR-Spectroscopy

Additionally, the samples were measured near infrared (NIR) spectroscopically with the integrating sphere module at 6 different sample positions in diffuse reflection. The spectra were recorded with a MPA^TM^ FT-NIR spectrometer from Bruker Optik GmbH. The NIR-range covers the wavenumber range from 12,500 to 4000 cm^−1^ and is characteristic for overtone and combination oscillations of e.g., CH-, OH-, and NH- groups. The measurements of the samples were performed in transmission at a resolution of 16 cm^−1^ and a scan number of 64. The relevant NIR-range for the analysis were the range 7300 to 4020 cm^−1^. A partial least squares (PLS) regression of spectroscopic data and corresponding concentration values of the individual components was used to create a calibration model. The basis of the calibration work was a set of reference samples with defined concentrations.

The PLS-model is improved by changing the frequency range, mathematical data pre-treatment (first derivation), selecting the appropriate number of factors and automatic outlier detection. Here, the factor number is equal to the number of PLS-factors. The concentration and spectral data matrix were decomposed into scores and loading vectors by the PLS algorithm. The model created in each case was validated by the chemometric method.

### 4.5. Spectrophotometric Measurement with Data Color

The matcha and iron(II)-lactate hair tresses were measured spectrophotometrically using a Datacolor SF600 Spectraflash device. A D65 illuminant and diffuse/8° optical configuration were used for the spectrophotometer measurements. The spectral reflectance data for each sample from 380 to 700 nm were converted into colorimetric data using the DCI Color software. Reflectance measurements were recorded for each hair sample using an average of 4 measurements.

In 1976, CIE introduced the CIE-Lab three-dimensional color space. An important reason for the introduction was to standardize formulas for measuring color differences. Color distances should correspond as closely as possible to the perceived differences. Thus, the system is intended to create visual uniformity in the evaluation of color differences [12].

Hair color is expressed in terms of the L*, a*, b* color coordinates, where L* corresponds to the lightness (0 = black, 100 = white), a* to the red-green coordinate (−delta e green, +delta e red), and b* to the yellow-blue coordinate (−delta e = blue, +delta e = yellow) of a sample identifies a point in the 3D CIELAB color space that describes the color of the hair sample [13]. The color changes after each wash cycle were measured as ΔE. Absolute color differences are measured with the following, Euclidean color distance formula calculated as described in DIN 6174 as in formula (1) [14]:

(1) $$\Delta E=\sqrt{{\;\Delta L}^{2}+{\Delta a}^{2}\;+{\Delta b}^{2}}\;.$$

### 4.6. Statistical Analysis

All data analyses were conducted using the software Statistica 13.0 (StatSoft Inc., Tulsa, OK, USA). One-way ANOVA and post-hoc Tukey test were conducted if the dataset was normal distributed and homogeneity of variance was given. If one of the abovementioned assumptions is not fulfilled the nonparametric Kruskal–Wallis test and afterwards the Dwass–Steel–Critchlow–Fligner test is conducted. All results are presented as box–whisker plots. The box defined by the mean ± standard error (SE) and the whiskers represent the mean ±1.96* standard error, which give the limits of the confidence interval on 95% confidence level.

## 5. Patents

Patent resulting from this manuscript with the following Number: DE102020214790.6.

## Figures and Tables

**Table 1:** Color coordinates, L, a, b, and ΔE of hair tresses dyed with different concentrations of matcha in combination with 1 wt% iron(II)-lactate (n = 10). SD: standard deviation.

Concentration of Matcha					
Color Coordinates	0 SD	1 SD	10 SD	20 SD	30 SD
L	76.98 ± 0.41	49.33 ± 0.87	42.22 ± 1.15	40.81 ± 1.24	41.15 ± 0.72
a	0.42 ± 0.12	0.81 ± 0.1	1.1 ± 0.12	1.18 ± 0.11	1.07 ± 0.08
b	19.51 ± 0.54	2.75 ± 0,47	0.1 ± 0.42	−0.37 ± 0.46	−0.98 ± 0.42
ΔE	2.01 ± 0.55	31.23 ± 0.88	38.76 ± 1.18	40.23 ± 1.27	40.2 ± 0.82

**Table 2:** Color coordinates, L, a, b, and ΔE of hair tresses dyed with different concentrations of iron(II)-lactate in combination with 10 wt% matcha (n = 10). SD: standard deviation.

Concentration of Iron(II)-Lactate					
Color Coordinates	0 SD	0.001 SD	0.010 SD	0.1 SD	1 SD
L	76.60 ± 0.74	62.67 ± 1.41	43.56 ± 0.74	43.1 ± 0.81	70.17 ± 0.86
a	0.41 ± 0.23	0.57 ± 0.14	1.1 ± 0.06	0.94 ± 0.1	2.05 ± 0.3
b	18.08 ± 0.71	7.51 ± 1.05	−0.84 ± 0.3	−1.16 ± 0.42	18.09 ± 0.75
ΔE	1.24 ± 0.62	18.28 ± 1.72	38.92 ± 0.73	39.47 ± 0.76	7.55 ± 0.88

**Table 3:** Color coordinates, L, a, b, and ΔE of hair tresses dyed with commercial hair color Igora Royal Silverwhite Dove Grey (n = 5). SD: standard deviation.

Number of Hair Washes of Dove Grey					
Color Coordinates	6 SD	12 SD	18 SD	24 SD	30 SD
L	45.59 ± 0.37	49.47 ± 0.93	49.6 ± 1.2	50.01 ± 0.76	50.28 ± 1.12
a	1.4 ± 0.08	1.7 ± 0.1	1.69 ± 0.15	1.72 ± 0.09	1.84 ± 0.05
b	5.7 ± 0.24	7.59 ± 0.19	7.89 ± 0.22	8.26 ± 0.31	8.31 ± 0.29
ΔE	5.92 ± 0.4	10.24 ± 0.8	10.49 ± 1.08	11.01 ± 0.64	11.2 ± 1.03

**Table 4:** Color coordinates, L, a, b, and ΔE of hair tresses dyed with iron(II)-lactate in combination with 10 wt% matcha (n = 5). SD: standard deviation.

Number of Hair Washes of Matcha and Iron(II)-lactate					
Color Coordinates	6 SD	12 SD	18 SD	24 SD	30 SD
L	44.09 ± 2.12	48.03 ± 1.82	48.83 ± 1.3	49.36 ± 1.51	51.03 ± 1.31
a	0.93 ± 0.07	1.48 ± 0.13	1.51 ± 0.12	1.71 ± 0.78	1.53 ± 0.06
b	3.53 ± 0.76	7.05 ± 0.41	7.7 ± 0.37	8.54 ± 0.22	8.98 ± 0.36
ΔE	4.35 ± 1.65	7.34 ± 1.64	8.35 ± 1.14	9.92 ± 1.28	10.88 ± 1.25

## Data Availability

The data presented in this study are available on request from the corresponding author.

## Sample Availability

Samples of the compounds are available from the authors.
